# Identification of KIF11 as a Novel Target in Meningioma

**DOI:** 10.3390/cancers11040545

**Published:** 2019-04-15

**Authors:** Gerhard Jungwirth, Tao Yu, Mahmoud Moustafa, Carmen Rapp, Rolf Warta, Christine Jungk, Felix Sahm, Steffen Dettling, Klaus Zweckberger, Katrin Lamszus, Christian Senft, Mario Loehr, Almuth F. Keßler, Ralf Ketter, Manfred Westphal, Juergen Debus, Andreas von Deimling, Matthias Simon, Andreas Unterberg, Amir Abdollahi, Christel Herold-Mende

**Affiliations:** 1 Division of Experimental Neurosurgery, Department of Neurosurgery, University of Heidelberg, INF 400, D-69120 Heidelberg, Germany; gerhard.jungwirth@med.uni-heidelberg.de (G.J.); tao.yu@med.uni-heidelberg.de (T.Y.); carmen.rapp@med.uni-heidelberg.de (C.R.); rolf.warta@med.uni-heidelberg.de (R.W.); christine.jungk@med.uni-heidelberg.de (C.J.); steffen.dettling@gmail.com (S.D.); klaus.zweckberger@med.uni-heidelberg.de (K.Z.); andreas.unterberg@med.uni-heidelberg.de (A.U.); H.Mende@med.uni-heidelberg.de (C.H.-M.); 2 Department of Radiation Oncology, University of Heidelberg, INF 400, D-69120 Heidelberg, Germany; Mahmoud.Moustafa@med.uni-heidelberg.de (M.M.); Juergen.Debus@med.uni-heidelberg.de (J.D.); Amir.Abdollahi@med.uni-heidelberg.de (A.A.); 3 Department of Clinical Pathology, Suez Canal University, 41522 Ismailia, Egypt; 4 Department of Neuropathology, Institute of Pathology, University of Heidelberg, German Cancer Consortium, CCU Neuropathology, German Cancer Research Center, INF 224, D-69120 Heidelberg, Germany; Felix.Sahm@med.uni-heidelberg.de (F.S.); Andreas.vonDeimling@med.uni-heidelberg.de (A.v.D.); 5 Department of Neurosurgery, University Medical Center Hamburg Eppendorf, Martinistr 52, D-20246 Hamburg, Germany; lamszus@uke.uni-hamburg.de (K.L.); westphal@uke.de (M.W.); 6 Department of Neurosurgery, University Hospital, Goethe University, Schleusenweg 2, D-60528 Frankfurt am Main, Germany; c.senft@med.uni-frankfurt.de; 7 Department of Neurosurgery, University Hospital Wuerzburg, Josef-Schneider-Str 2, D-97080 Wuerzburg, Germany; loehr_m1@ukw.de (M.L.); kessler_a1@ukw.de (A.F.K.); 8 Department of Neurosurgery, Saarland University, Medical School, Kirrberger Str, D-66421 Homburg, Germany; Ralf.Ketter@uks.eu; 9 Department of Neurosurgery, Bethel Clinic, Burgsteig 13, D-33617 Bielefeld, Germany; matthias.simon@evkb.de

**Keywords:** meningioma, KIF, kinesin, KIF11, NCH93

## Abstract

Kinesins play an important role in many physiological functions including intracellular vesicle transport and mitosis. The emerging role of kinesins in different cancers led us to investigate the expression and functional role of kinesins in meningioma. Therefore, we re-analyzed our previous microarray dataset of benign, atypical, and anaplastic meningiomas (*n* = 62) and got evidence for differential expression of five kinesins (KIFC1, KIF4A, KIF11, KIF14 and KIF20A). Further validation in an extended study sample (*n* = 208) revealed a significant upregulation of these genes in WHO°I to °III meningiomas (WHO°I *n* = 61, WHO°II *n* = 88, and WHO°III *n* = 59), which was most pronounced in clinically more aggressive tumors of the same WHO grade. Immunohistochemical staining confirmed a WHO grade-associated upregulated protein expression in meningioma tissues. Furthermore, high mRNA expression levels of KIFC1, KIF11, KIF14 and KIF20A were associated with shorter progression-free survival. On a functional level, knockdown of kinesins in Ben-Men-1 cells and in the newly established anaplastic meningioma cell line NCH93 resulted in a significantly inhibited tumor cell proliferation upon siRNA-mediated downregulation of KIF11 in both cell lines by up to 95% and 71%, respectively. Taken together, in this study we were able to identify the prognostic and functional role of several kinesin family members of which KIF11 exhibits the most promising properties as a novel prognostic marker and therapeutic target, which may offer new treatment options for aggressive meningiomas.

## 1. Introduction

Meningiomas (MGMs) are tumors of the central nervous system and account for one-third of all brain tumors. They are assumed to arise from the meningeal coverings of the brain and the spinal cord [1]. Meningiomas are histologically classified according to the World Health Organization’s (WHO) grading scheme [2]. Nearly 80% of meningiomas are benign, corresponding to WHO°I, 10–15% are atypical (WHO°II), and only 2–5% are anaplastic meningiomas WHO°III. Their incidence ranges from 1.3 per 100,000 to 7.8 per 100,000 [3]. Clinical outcome strongly depends on the WHO grade. While patients with benign meningiomas have five-year survival rates of 92%, it decreases in atypical meningiomas to 78%, and in patients with WHO°III meningiomas, survival rates drop down to 47% [4,5,6,7]. Therapy options include observation and maximal safe resection. Residual or recurring tumors are usually treated with conventional radiotherapy while all tested chemotherapeutic agents failed to show any significant effect in the treatment of aggressive meningiomas [8].

Recent studies revealed distinct but in part mutually exclusive genetic alterations in meningiomas including NF2, TRAF7, AKT1, SMO, KLF4, POLR2A, PIK3CA and TERT promoter mutations [9,10,11,12,13,14]. The identification of these mutations led to the investigation of inhibitors targeting proteins such as AKT1, SMO, mTOR and FAK [15,16,17]. Based on these findings, several clinical trials have been initiated (NCT02523014; NCT03071874; NCT02831257; and EORTC-1320).

The kinesin superfamily (KIF) members are highly conserved motor proteins, which are subdivided into 14 families (Kinesin 1–14A/B) [18,19,20]. Their motor domain enables binding and stepping across microtubules by converting the chemical energy of ATP in mechanical ATP hydrolysis into a mechanical force [21,22]. KIF proteins play an important role in cellular functions including mitosis and intracellular transport of vesicles and organelles [22,23]. Due to overexpression of certain motor kinesins such as KIF11, additional outward forces can be generated during mitosis leading to premature separation of sister chromatids and an unequal distribution of chromosomes and thus may cause aneuploid daughter cells [22,24]. The resulting genetic instability can cause progression of cancer, for example through an increased invasion and the development of metastasis [25]. Accordingly, kinesin proteins have been shown to be upregulated and may be associated with a reduced progression-free survival in several cancer types including colorectal cancer [26], melanoma [27], retinoblastoma [28], breast [29], lung [30], pancreatic [31], laryngeal [32], hepatocellular [33], ovarian carcinoma and glioma [34,35,36]. However, data on the functional expression of kinesins in meningiomas are missing.

In the present study, we identified a WHO-dependent upregulated mRNA expression of five members of the kinesin family by re-analyzing a previous transcriptome dataset of meningiomas [37]. Their differential expression could be confirmed at the mRNA and protein level. In addition, in the presence of higher mRNA levels, we could demonstrate a poorer survival for several of the kinesin family members. However, only knockdown of KIF11 resulted in a markedly reduced tumor cell proliferation in Ben-Men-1 cells and the newly established anaplastic NF2-mutated meningioma cell line NCH93, proposing KIF11 as a novel prognostic marker and therapeutic target.

## 2. Results

### 2.1. Kinesin mRNA Levels Increase with WHO Grade of Meningiomas

**Figure 1:**
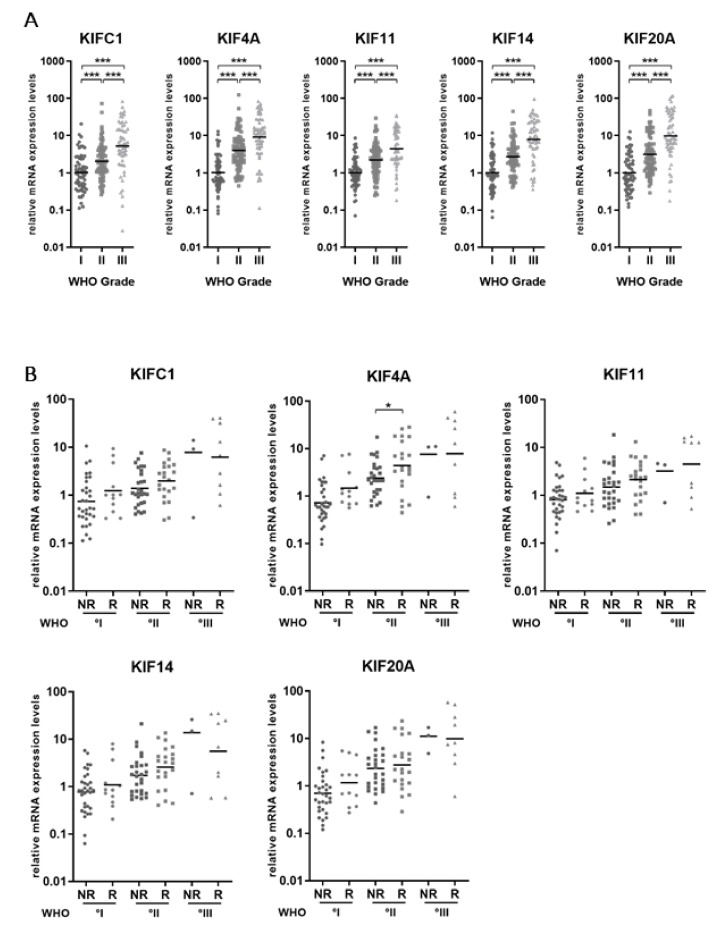
mRNA expression levels of kinesin family members increase with WHO grade in meningiomas. (**A**) qRT-PCR expression data for KIFC1, KIF4A, KIF11, KIF14A and KIF20A are shown. mRNA levels were normalized to the mean of the meningioma WHO°I samples (*n* = 61). (**B**) Kinesin mRNA expression according to the future clinical course of the disease. Primary meningioma tissue samples were grouped into non-recurrent (NR) (follow-up period ≥ 60 months), and clinically aggressive tumors presenting with recurrent meningiomas during the observation time (R) of each WHO grade. Significance levels: * *p* < 0.05, *** *p* < 0.001.

In a previous study, we assessed transcriptome microarray data of 62 meningiomas including a substantial number of rare higher-grade tumors (WHO°I *n* = 20, WHO°II *n* = 14 and WHO°III *n* = 28) [37]. By re-analyzing our dataset for the expression of members of the kinesin family, we identified significant differential gene expression of five kinesins: KIFC1, KIF4A, KIF11, KIF14 and KIF20A (Supplementary Table S3) (fold change > 1.25 and *p* < 0.01). We next sought to validate the differential expression of these genes in an extended set of meningioma tissue samples by quantitative real-time PCR (qRT-PCR). In total, we used RNA of 61 WHO°I, 88 WHO°II and 59 WHO°III meningiomas. Patient characteristics are presented in Table 1. In accordance with the microarray data, we observed a significant WHO grade-associated increase of all tested kinesin genes (*p* < 0.001; Figure 1A).

### 2.2. Subgroup Analysis Reveals Elevated KIF4A Levels in MGMs Experiencing Future Recurrence or Malignant Progression

Next, we were interested to find out if kinesin mRNA expression might predict the future clinical course of the disease in newly diagnosed primary tumors even within a given WHO grade. Since meningiomas tend to recur or even to progress to a higher grade, we divided our primary meningioma samples into tumors without future recurrence within the observed time interval (no recurrence NR, ≥60 months), and clinically aggressive tumors reoccurring during our observation period (recurrence R) (Figure 1B; Table 2). There was a general trend for a higher expression of all kinesins studied in more aggressive tumors of the same WHO grade. However, significant differences were only observed for KIF4A between 2NR (WHO grade II, no recurrence) and the more aggressive 2R (WHO°II, recurrence) tumors (*p* = 0.01).

### 2.3. WHO Grade-Specific Increase of Kinesin Protein Levels in Meningioma Tissue Samples

**Figure 2:**
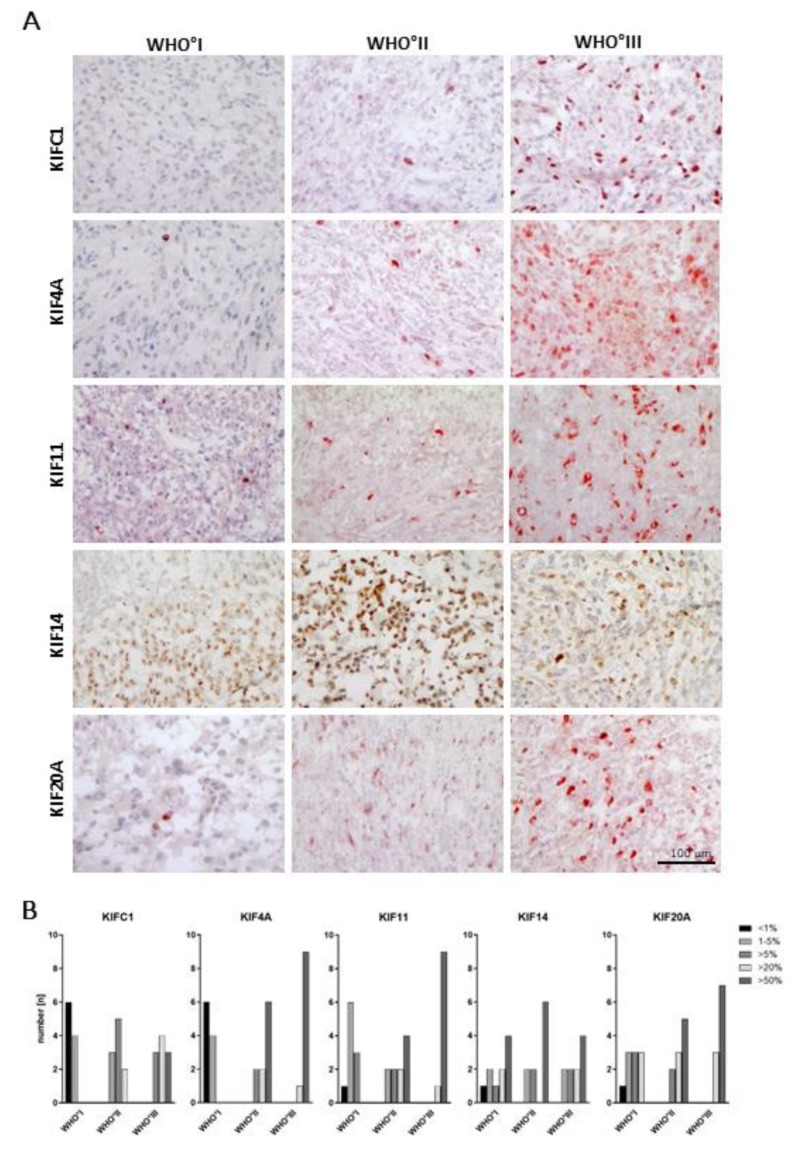
Immunohistochemical staining of meningioma tissue samples reveals a WHO grade-specific increase of protein levels. (**A**) Representative immunohistochemical stainings are shown for KIFC1, KIF4A, KIF14 and KIF20A and each WHO grade. Ten meningioma tissue samples of each WHO grade were stained with antibodies against KIFC1, KIF4A, KIF11, KIF14, or KIF20A. (**B**) Staining was evaluated as percent positive cells in five categories (<1%, 1–5%, >5%, >20% and >50% positive cells).

To learn more about the tissue distribution of kinesin expression on a cellular level, we studied the protein expression in meningioma tissues by immunohistochemistry (Figure 2). Therefore, we used ten meningioma tissue samples for each WHO grade and stained them for KIFC1, KIF4A, KIF11, KIF14 and KIF20A. Patient data are depicted in Supplementary Table S2. Staining was evaluated as percent positive cells in five categories (<1%, 1–5%, >5%, >20% and >50%). Staining results are illustrated in Figure 2.

In general, we observed a heterogeneous predominantly perinuclear staining pattern for all kinesin members. For instance, 60% of WHO°I tumors showed less than 1% of KIFC1-positive cells, while in the remaining tumors up to 5% of cells were stained. In contrast, in WHO°II tumors most of the samples were positive for KIFC1 in up to 20% with a further increase in the majority of WHO°III tumors. Regarding KIF4A and KIF11, we made similar observations, though the number of meningiomas with more than 50% kinesin-expressing cells further increased in WHO°II and °III tumors. In contrast, protein expression of KIF14 did not seem to vary according to the WHO grade, while for KIF20A, a WHO grade-dependent increased expression was visible again.

In summary, except for KIF14, all kinesins analyzed showed an increment in the number of positive cells with increasing WHO grade, which was most pronounced for KIF4A and KIF11. Thus, we could confirm that the observed differential mRNA expression translates to a differential protein expression for most of the kinesin family members tested.

### 2.4. High Levels of KIFC1, KIF11, KIF14 and KIF20A Are Associated with Shorter Progression-Free Survival

**Figure 3:**
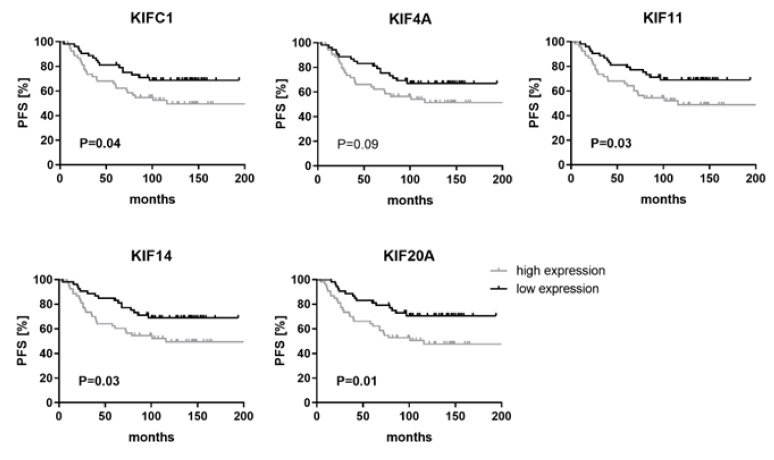
High mRNA expression levels of KIFC1, KIF11, KIF14 and KIF20A are associated with shorter progression-free survival. Kaplan–Meier plots show survival association of each investigated kinesin. The patients were categorized into two groups according to their median mRNA expression levels into high (grey curve) and low (black curve) expression. Only primary tumor samples and Simpson °I–III resected tumors were included in this analysis (*n* = 106). Prognostic significance was determined using Log-rank (Mantel–Cox) tests. PFS: Progression-free survival.

Next, we explored if mRNA expression levels of kinesin genes are associated with clinical outcome. To avoid a survival bias due to incomplete tumor resection or treatment-induced expression changes in recurrent tumors, only patients who underwent a gross total tumor resection (Simpson °I–III), a minimal follow-up time of 60 months, and without any prior treatment were included (*n* = 106) (Table 2). MicroRNA expression was divided by the median in a low and high expression group to assess progression-free survival of patients in these two groups as illustrated by Kaplan–Meier plots (Figure 3).

This analysis revealed that high levels of KIFC1 (*p* = 0.04; HR 1.84), KIF11 (*p* = 0.03; HR 1.88), KIF14 (*p* = 0.03; HR 1.91) and KIF20A (*p* = 0.01; HR 2.13) were associated with a shorter progression-free survival, suggesting kinesins as novel prognostic factors in meningiomas.

### 2.5. NCH93 as a Novel Anaplastic Meningioma Cell Line for Functional Testing

**Figure 4:**
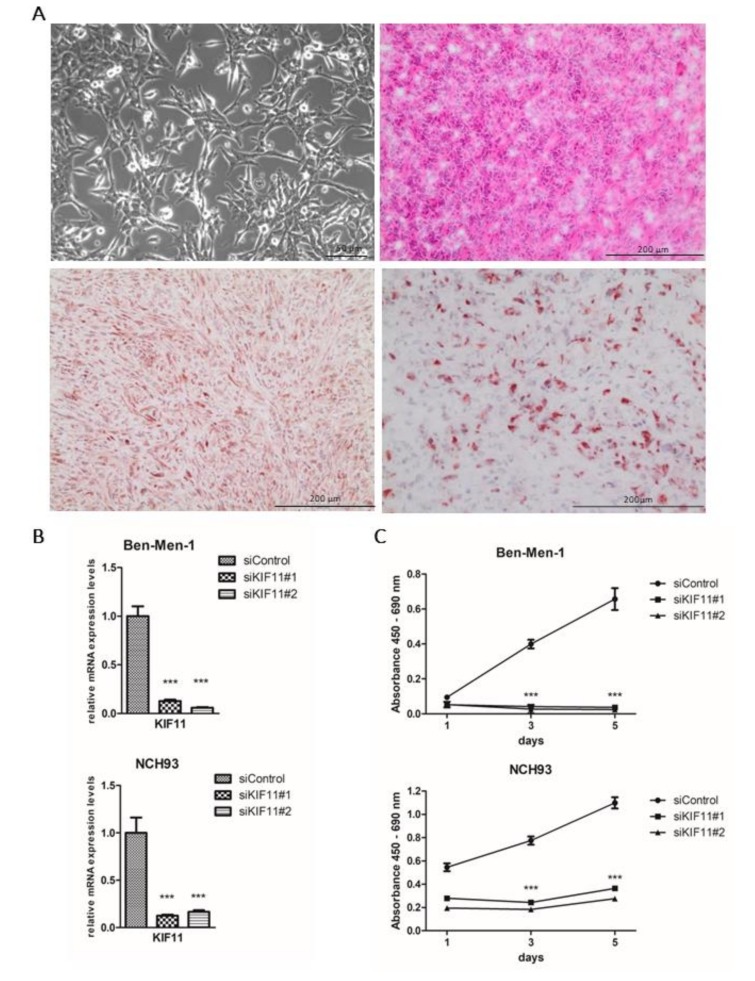
siRNA-mediated knockdown of KIF11 reduces the proliferation of Ben-Men-1 cells and the human anaplastic meningioma cell line NCH93. (**A**, left upper corner) Representative light microscopy images of adherently growing NCH93 cells. (**A**, right upper corner) Representative H&E staining of NCH93 tumors grown in the flanks of mice. (**A**, left and right bottom corners) Immunohistochemical staining for EMA and Ki67, respectively. (**B**) Transfection of Ben-Men-1 and NCH93 cells with 25 nM corresponding siRNAs overnight. Knockdown efficiency was evaluated by qRT-PCR and data were normalized to the siRNA of the control sample. (**C**) BrdU proliferation assay was performed on day 1, day 3 and day 5 after seeding. Error bars indicate SEM. Significance levels: *** *p* < 0.001.

Due to a paucity of well-growing human in vitro and in vivo meningioma models, we established the novel meningioma cell line NCH93. Cell culture was established from a human anaplastic meningioma as described [38] (Figure 4A).

To confirm their tumorigenicity, 10^6^ NCH93 cells embedded in Matrigel were injected in the flanks of 5–6 week-old female NMRI/nu mice. Five out of six tumors reached a size of approximately 200 mm^3^ five weeks after implantation, and after two additional weeks, the mice were sacrificed reaching a tumor volume of approximately 1000 mm^3^. To further characterize the cell line, we performed a targeted panel sequencing. This panel contained 130 genes frequently mutated in brain tumors including MGMs. Sequencing revealed a deleterious NF2 frameshift deletion (p.D130fs, p.D172fs, p.D213fs) and pathogenic missense mutation in the genes ALK (p.K1491R) and PTCH1 (p.P1164L) according to COSMIC data (https://cancer.sanger.ac.uk/cosmic/browse/genome) (Supplementary Table S4). Excised tumors were immunohistochemically stained for the meningioma marker EMA and the proliferation marker Ki67. NCH93 tumor tissue exhibited a strong staining for EMA and a substantial number of about 50% of cells stained positively for Ki67, which is in concordance with its fast-paced in vivo tumor growth.

### 2.6. Depletion of KIF11 via siRNA Inhibits Meningioma Proliferation In Vitro

To finally address the question, if differentially expressed kinesin family members have an impact on tumor cell proliferation of meningioma cells, we performed a siRNA-mediated knockdown of KIFC1, KIF4A, KIF11, KIF14 and KIF20A. To this end, we transfected the benign meningioma cell line Ben-Men-1 and the anaplastic meningioma cell line NCH93 with two different siRNAs targeting each kinesin and a negative control siRNA and analyzed the proliferative activity by the BrdU assay at different points of time. siRNA-mediated knockdown resulted in >70% knockdown on the mRNA level of all targeted genes as validated by qRT-PCR (Figure 4B; Supplementary Figure S1). Subsequent BrdU incorporation assay revealed a significant decrease in tumor cell proliferation in KIF11-depleted Ben-Men-1 and NCH93 cells for both siRNAs employed (Figure 4C). Cell proliferation was inhibited by siRNA against KIF11 in the benign cell line Ben-Men-1 by 91% and 95% on day 3 and 5 (*p* < 0.001) and by 56%, 72% and 71% on day 1, 3 and 5 in the malignant cell line NCH93 (*p* < 0.001), respectively. Moreover, knockdown of KIF4A also resulted in reduced cell proliferation (*p* < 0.001) of Ben-Men-1 cells, but not of NCH93 cells. Knockdown of other kinesins failed to affect tumor cell proliferation in any of the cell lines (Supplementary Figure S2). In summary, these data support an important functional role of KIF11 for meningioma growth and its use as a novel therapeutic target in clinically aggressive meningiomas.

## 3. Discussion

Clinically aggressive meningiomas are still challenging due to the lack of therapy options despite surgery and radiotherapy [8]. Since effective chemotherapeutic agents are still missing, the identification of novel treatment approaches is urgently needed. To query if kinesin family members might represent novel molecular therapeutic targets, we re-analyzed our previous microarray data set of benign, atypical and anaplastic meningiomas and identified five differentially expressed kinesins (KIFC1, KIF4A, KIF11, KIF14 and KIF20A). This is of major interest because kinesins play an important role in many physiological processes including intracellular vesicle transport and mitosis and are upregulated in many different cancer types [18,39]. Further validation in an extended study sample of 208 cases fairly representing all WHO grades revealed a significant upregulation of all investigated kinesins in meningiomas WHO°I to °III which even translated into an upregulated protein expression. As a further indication for the importance of kinesin family members for meningioma growth, we found that high mRNA expression levels of almost all kinesins were associated with shorter progression-free survival. To further elaborate on the functional role of kinesins, we performed knockdown experiments on Ben-Men-1 cells and the newly developed anaplastic meningioma cell line NCH93. This resulted in a significantly inhibited tumor cell proliferation of KIF11-depleted cells by up to 95% and 71%, respectively, suggesting KIF11 as a promising novel therapeutic target in meningiomas.

KIF11′s functions can be divided into mitosis-related and non-mitosis-related properties. The gene is essential to assemble the mitotic spindle, and its loss leads to a monopolar spindle phenotype due to the failure of centrosome separation, resulting in a radial array of microtubules with the chromosomes distributed along the circumference. This causes a G2/M arrest, which may induce apoptosis [24,40]. Non-mitosis-related functions of KIF11 include neuronal growth cone extension, navigation, and migration [41]. Our demonstration that KIF11 mRNA and protein levels increased with meningioma WHO grade and that higher mRNA levels of KIF11 were related to shorter progression-free survival, is consistent with prior literature reports in other cancer types [24,42,43,44,45,46]. Although all kinesins resulted in a WHO grade-dependent upregulation, only the knockdown of KIF11 with two different siRNAs targeting KIF11 led to an inhibited proliferation of the benign meningioma cell line Ben-Men-1 and the anaplastic NCH93 cell line in vitro, indicating a pivotal role in tumorigenesis and the sustained growth of meningiomas. Interestingly, several selective small molecule inhibitors have been developed and pre- and clinically tested with promising results in other tumors types, suggesting their future analysis in meningiomas [47,48,49,50,51,52,53,54,55,56,57,58].

KIFC1 plays an important physiological role in the vesicular and organelle trafficking, spermiogenesis and oocyte development [59,60,61,62,63]. Furthermore, KIFC1 is widely expressed in cancer cells and may be essential for the bi-multipolar mitotic division of cancer cells but is dispensable in ordinary somatic cells, which qualifies KIFC1 as an excellent target in cancer therapy [29,64,65,66,67,68,69,70]. Direct KIFC1 inhibitors including AZ82, CW069 and transcription inhibitor PJ34 are readily available and have shown promising results in vitro and in vivo [68]. We demonstrated that KIFC1 mRNA and protein expression increased in meningiomas with the WHO grade. However, although progression-free survival was related to the expression of KIFC1 mRNA, the knockdown of KIFC1 in two different meningioma cell lines did not show any effect on proliferation, indicating KIFC1 as a dispensable gene for meningioma proliferation which might be due to the upregulation of currently unknown compensatory genes [29,65,67,68,69,70,71,72].

KIF4A, KIF14 and KIF20A are shown to be overexpressed across many human cancer types suggesting a link between expression levels and overall survival. Furthermore, their knockdown inhibited proliferation and migration in breast, prostate, lung, ovarian and gastric tumors [73,74,75,76,77,78,79,80,81,82,83,84,85]. In meningiomas, however, KIF20A demonstrated an effect on progression-free survival but failed to show a significant functional impact on cell proliferation in vitro. KIF4A showed a differential protein and mRNA expression and discrimination of non-recurrent WHO°II meningiomas vs. clinically aggressive tumors of the same grade. In addition, high mRNA levels of KIF4A showed a trend towards a poorer progression-free survival (*p* = 0.09) and upon knockdown, reduced the proliferation of Ben-Men-1 cells. Accordingly, KIF4A could still serve as a target in meningiomas and deserves further investigation. However, small molecule inhibitors are currently not available. Last but not least as for KIF14, despite differential protein and mRNA expression in meningiomas, progression-free survival or proliferation in vitro seemed to be unaffected by KIF14. Thus, as compared to the strong effects seen for other kinesin family members, KIF14 may play a minor role in meningioma pathogenesis.

Finally, appropriate meningioma in vitro and in vivo models are rare. The Ben-Men-1 cell line was established from a benign meningioma, which has been genetically modified by retrovirally introducing the hTERT gene and exhibits an unusual high proliferation rate for a WHO°I meningioma [86]. If at all, it exhibits only a slow growth rate in vivo [86,87]. Other cell lines are less commonly used or are quite old [87]. This forced us to establish a novel anaplastic meningioma cell line, which shows unlimited growth in vitro and after xenotransplantation forms tumors within 5–6 weeks in the flank of NMRI/nu mice. Panel sequencing of targeted genes revealed a deleterious NF2 frameshift mutation and pathogenic PTCH1 and ALK mutations. Therefore, this novel cell line might accelerate meningioma research by providing a platform for functional in vitro and in vivo experiments.

Altogether, in this study, we were able to characterize the prognostic and functional role of several kinesin family members of which KIF11 provided the most promising properties as a novel prognostic marker and therapeutic target, which may offer new treatment options for aggressive meningiomas.

## 4. Materials and Methods

### 4.1. Tumor Samples and RNA Isolation

Patients and corresponding tumors were included as part of the FORAMEN effort of the Neuro-Oncology Section of the German Society of Neurosurgery (DGNC) [37]. FORAMEN aims at conducting clinical and translational projects on aggressive meningiomas. The study was approved by the institutional review board at Heidelberg Medical Faculty (Ethical code: S-005/2003, permission date: 31 March 2003). Informed consent was received from all patients. Fresh tumor material obtained intraoperatively was snap-frozen and stored at −80 °C until further processing. Only samples with a vital tumor cell content >60% as determined on H&E-stained slides from each tissue by a board-certified neuropathologist were eligible (Department of Neuropathology, University Hospital Heidelberg, Germany). Total RNA was extracted from tissues using the AllPrep Kit (Qiagen) according to the manufacturer’s instructions. RNA integrity was assessed by the Agilent 2100 Bioanalyzer. RNA was quantified by NanoDrop ND-1000 spectrophotometer (Thermo-Scientific, Waltham, MA, USA), and then stored at −80 °C until further analysis.

### 4.2. Quantitative Real-Time PCR

Equal amounts of total RNA (1 µg) were reverse-transcribed using the Transcriptor First Strand cDNA Synthesis Kit (Roche, Basel, Switzerland) with random hexamer primers for one hour at 50 °C. qPCR was performed in triplicates on a LightCycler 480 (Roche) using the LightCycler 480 Probes Master and probes from the Universal Probe Library (Roche) as described (www.roche-applied-science.com). Relative fold changes between the expression of target genes were calculated by using the 2^-ΔΔCq method. GAPDH and ACTB were used as reference genes. The relative expression levels of KIFC1, KIF4A, KIF11, KIF14A and KIF20A mRNA levels were normalized to the mean of the meningioma WHO°I samples. The primers used are shown in Supplementary Table S1.

### 4.3. Immunohistochemical Staining

Thirty tissue samples of meningioma patients were used (WHO°I *n* = 10, WHO°II *n* = 10, WHO°III *n* = 10, Supplementary Table S2). Stainings were performed on acetone-fixed cryosections (5–7 µm). Antibodies and dilutions used were as follows: anti-KIFC1 (rabbit monoclonal IgG, ab172620, Abcam, Cambridge, UK) 1:100, anti-KIF4A (rabbit polyclonal IgG, ab122227, Abcam) 1:400, anti-Eg5/KIF11 (rabbit polyclonal IgG, ab37814, Abcam) 1:1000, anti-KIF14 (rabbit polyclonal IgG, ab71155, Abcam) 1:250 (using UltraVision Quanto Detection Kit HRP, Thermo Scientific, Waltham, MA, USA), and anti-KIF20A (rabbit polyclonal IgG, ab85644, Abcam) 1:400. Primary antibodies were diluted with DAKO diluent (Agilent, Santa Clara, CA, USA) in the above-mentioned concentrations and then stained for 60 min at room temperature (RT) covered from light. Slides were then washed three times with PBS-T (0.05%). Next, secondary antibody (anti-rabbit, PK-6101, Vectastain, Vector labs, Burlingame, CA, USA) diluted in serum and DPBS (ThermoFisher Scientific) were added to the slides and incubated for 30 min. Then the slides were incubated with an avidin-biotin-complex (PK-6101, Vectastain, Vector labs) for 30 min, followed by a PBS wash and an AEC substrate incubation. The slides were then rinsed with distilled water for five min and counterstained with hematoxylin. Four pictures of each slide were taken (three pictures at 20× and one at 10× magnification) and two independent observers evaluated each tissue sample according to the following categories: <1%, 1–5%, >5%, >20% and >50% positive cells. Rabbit IgGs served as a negative control (anti-rabbit, PK-6101, Vectastain, Vector labs). NCH93 tumor samples excised from mice were stained accordingly following the same protocol as mentioned above. Antibodies and dilutions for NCH93 tumor staining were used as follows: anti-EMA (rabbit monoclonal IgG, ab136615, Abcam) 1:50 and anti-Ki67 (rabbit monoclonal IgG, ab15580, Abcam) 1:50.

### 4.4. Cell Lines

NCH93 tumor cells originated from a 64-year-old man suffering from a relapsed anaplastic meningioma (WHO°III) located in the left parieto-occipital region. The tumor sample was first mechanically dissected. Then the resulting cell suspension was cultured in Dulbecco’s minimal Eagle’s medium supplemented with 10% fetal calf serum (Sigma-Aldrich, St. Louis, MO, USA), 2% L-GlutaMAX (Sigma-Aldrich) and 1% Penicillin/Streptomycin (Sigma-Aldrich). Cells were subcultured in a ratio of 1:10 twice a week. *Mycoplasma* contamination was excluded by 4′,6-diamidino-2-phenylindole staining (Roche). The benign cell line Ben-Men-1 was purchased from DSMZ (Braunschweig, Germany). Cells were grown in the same medium as NCH93, and both were cultured at 37 °C in a humidified environment in a 5% CO_2_ atmosphere. Cell lines were authenticated by STR DNA profiling analysis (Leibniz Institute DMSZ, Braunschweig, Germany).

### 4.5. Panel Sequencing

NCH93 cells were profiled by targeted panel sequencing to identify meningioma-typical aberrations. The panel contained 130 genes reported to be frequently mutated in brain tumors including MGMs as described previously [88]. Sequencing was done by applying a custom hybrid capture approach (Agilent Technologies, CA, USA) on a NextSeq500 instrument (Illumina, San Diego, CA, USA). For single nucleotide variants and InDel calling, SAMtools mpileup and Platypus were utilized. The panel was designed to assess the frequency of known brain tumor mutations rather than novel mutational events.

### 4.6. siRNA-Mediated Knockdown

In brief, meningioma cells were transfected with siRNA against KIFC1 (s7906, s7907, Invitrogen, Carlsbad, CA, USA), KIF4A (s24407, s24408, Invitrogen), KIF11 (s7902, s7903, Invitrogen), KIF14 (s19263, s19265, Invitrogen) and KIF20A (s19676, s19677, Invitrogen) or siRNA negative control (Cat. # 4390843, Invitrogen). Lipofectamine RNAiMAX reagent (Cat. # 13778030, Invitrogen) was used to deliver siRNAs into the cells. Cells were transfected with a final concentration of 25 nM siRNA and 7.5 µL Lipofectamine RNAiMAX in 1 mL OptiMEM (Gibco) overnight. On the next day, the medium was changed to DMEM supplemented with 10% FCS, 2% GlutaMAX (Gibco), and 1% Pen/Strep and cells were further used for experiments. Validation of the knockdown was assessed by qRT-PCR. A decrease in mRNA levels of >70% compared to siRNA negative control was used as a threshold for a successful siRNA-mediated knockdown of the target gene.

### 4.7. Cell Proliferation Assay

Cell proliferation was quantified using the Cell Proliferation ELISA, BrdU Kit (). NCH93 and Ben-Men-1 cells were cultured in 6-well plates at a density of 3 × 10^5^ cells/well and transfected as described above. The day after, cells were harvested and counted, and 5000 cells were seeded in 96-well plates in 100 µL medium. On day 1, 3 and 5, 10 µL BrdU labeling solution was added to the cells (final concentration of 10 µM BrdU) and incubated for two hours. Next, the medium was removed from the cells by tapping off. Cells were fixed with 200 µL per well FixDenat and incubated for an additional 30 min at RT. FixDenat was removed by tapping off and 100 µL anti-BrdU-POD working solution per well was added and incubated for 90 min at RT. The antibody conjugate was removed and wells were rinsed three times with 200 µL PBS. Thereafter, 100 µL substrate solution was added and incubated for an additional 10 min at RT. Then absorbance was measured at 450 nm and reference wavelength 690 nm by Microplate Reader Tecan Infinite PRO.

### 4.8. Xenograft Mouse Model

To further characterize the NCH93 cells, their tumorigenicity was tested in a xenograft mouse model. Animal experiments were approved by the Regierungspraesidium Karlsruhe (35-9185.81/G-193/17). For the in vivo tumorigenicity study, 1 × 10^6^ cells suspended in 100 µL Matrigel (Cat. # 356237, Corning, New York, NY, USA) were injected subcutaneously into the flank regions of 5–6 week-old female NMRI/nu mice (Janvier Laboratory, Le Genest-Saint-Isle, France). Tumor growth was measured every second day by a caliper. Animals were sacrificed seven weeks after tumor cell injection reaching a tumor volume of approximately 1000 mm³.

### 4.9. Statistical Analysis

All measurements were performed in triplicates. Values are expressed as mean ± SEM; *p*-values were calculated using two-tailed Student’s *t*-test in GraphPad Prism (Version 8.0.1, GraphPad, San Diego, CA, USA). For survival analysis, progression-free survival (PFS) was used as an end-point. Progression was assessed in MRI scans. Prognostic significance was determined using the Log-rank tests. *p*-values < 0.05 were considered significant (* *p* < 0.05; ** *p* < 0.01; *** *p* < 0.001).

## 5. Conclusions

In a large set of benign, atypical and anaplastic meningiomas, several kinesin family members have been discovered as prognostic factors. Among them, knockdown experiments identified KIF11 as the most promising therapeutic target. Finally, by establishing and characterizing the NF2-mutated anaplastic meningioma cell line NCH93, we provide a novel powerful cell model for meningioma research. This may help to improve the treatment of aggressive meningiomas.

## Figures and Tables

**Table 1:** Clinical characteristics of the study sample used to validate the mRNA expression of kinesin family members (*n* = 208).

Clinical Factors	Group	Patients	
		*n*	(%)
**Sex**	Male	93	45
	Female	115	55
**Age** at 1st diagnosis (years)	Median	59	
	Range	18–87	
**WHO Grade**	WHO°I	61	29.3
	WHO°II	88	42.3
	WHO°III	59	28.4
**Subtype**	Fibroblastic	8	3.8
	Meningothelial	9	4.3
	Transitional	31	14.9
	Psammomatous	4	1.9
	Angiomatous	1	0.5
	Secretory	3	1.4
	Atypical	75	36.1
	Anaplastic	40	19.2
	Rhabdoid	2	1.0
	Papillary	2	1.0
	Mixed/Unknown	33	15.9
**Location**	Convexity	75	36.1
	Falx	32	15.4
	Tentorial or parasagittal	42	20.2
	Cranial base	48	23.1
	Multiple	4	1.9
	Other/NA	7	3.4
**Primary or recurrent tumor**	Primary tumor	134	64
	Recurrent tumor	74	36
**Resection grade**	Simpson °I	101	48.6
	Simpson °II	59	28.4
	Simpson °III	27	13
	Simpson °IV	18	8.7
	Simpson °V	1	0.5
	Unknown	2	1.0
**Postoperative treatment**	Radiotherapy	62	
	Chemotherapy	6	

**Table 2:** Clinical characteristics of untreated primary meningioma patients used for the survival analyses (*n* = 106).

Clinical Factors	Group	Patients	
		*n*	(%)
**Sex**	Male	39	36.8
	Female	67	63.2
**Age** at 1st diagnosis (years)	Median	60	
	Range	24–85	
**WHO Grade**	WHO°I	44	41.5
	WHO°II	50	47.2
	WHO°III	12	11.3
**Subtype**	Fibroblastic	7	6.6
	Meningothelial	7	6.6
	Transitional	20	18.9
	Psammomatous	4	3.8
	Angiomatous	0	0.0
	Secretory	3	2.8
	Atypical	42	39.6
	Anaplastic	9	8.5
	Rhabdoid	0	0.0
	Papillary	1	0.9
	Mixed/Unknown	13	12.3
**Location**	Convexity	38	35.8
	Falx	16	15.1
	Tentorial or parasagittal	22	20.8
	Cranial base	25	23.6
	Multiple	0	0.0
	Other/NA	5	4.7
**Primary or recurrent tumor**	Primary tumor	106	100
	Recurrent tumor	0	0
**Resection grade**	Simpson °I	59	55.7
	Simpson °II	33	31.1
	Simpson °III	14	13.2
**Future clinical progression**	No future recurrence	63	
	Recurrence with same WHO°	34	
	Recurrence with higher WHO°	9	
		**Median**	
**Follow-up time** (months)	WHO°I	135.5	
	WHO°II	100.1	
	WHO°III	47.9	

## Supplementary Materials

The following are available online at https://www.mdpi.com/2072-6694/11/4/545/s1, Figure S1: Efficiency of siRNA-mediated knockdown evaluated by qRT-PCR, Figure S2: Knockdown of KIFC1, KIF4A, KIF14 and KIF20A failed to show a robust effect in both meningioma cell lines, Table S1: Primer sequences, Table S2: Clinical data of meningiomas employed for protein expression analysis (n = 30), Table S3: Comparative expression of kinesin family members in a published microarray dataset, Table S4: Panel Sequencing data of NCH93 cells.
