# Supplementary material for: Identification of KIF11 as a Novel Target in Meningioma
Source: Cancers (Basel). 2019 Apr 15;11(4):545. doi: 10.3390/cancers11040545 (PMC6521001; doi:10.3390/cancers11040545)
Supplement: Supplementary file 1 [file cancers-11-00545-s001.pdf]

cancers

MDPI

Supplementary Materials: Identification of KIF11 As a Novel Target in Meningioma

Gerhard Jungwirth, Tao Yu, Mahmoud Moustafa, Carmen Rapp, Rolf Warta, Christine Jungk, Felix Sahm, Steffen Dettling, Klaus Zweckberger, Katrin Lamszus, Christian Senft, Mario Loehr, Almuth F. Keßler, Ralf Ketter, Manfred Westphal, Juergen Debus, Andreas von Deimling, Matthias Simon, Andreas Unterberg, Amir Abdollahi and Christel Herold-Mende

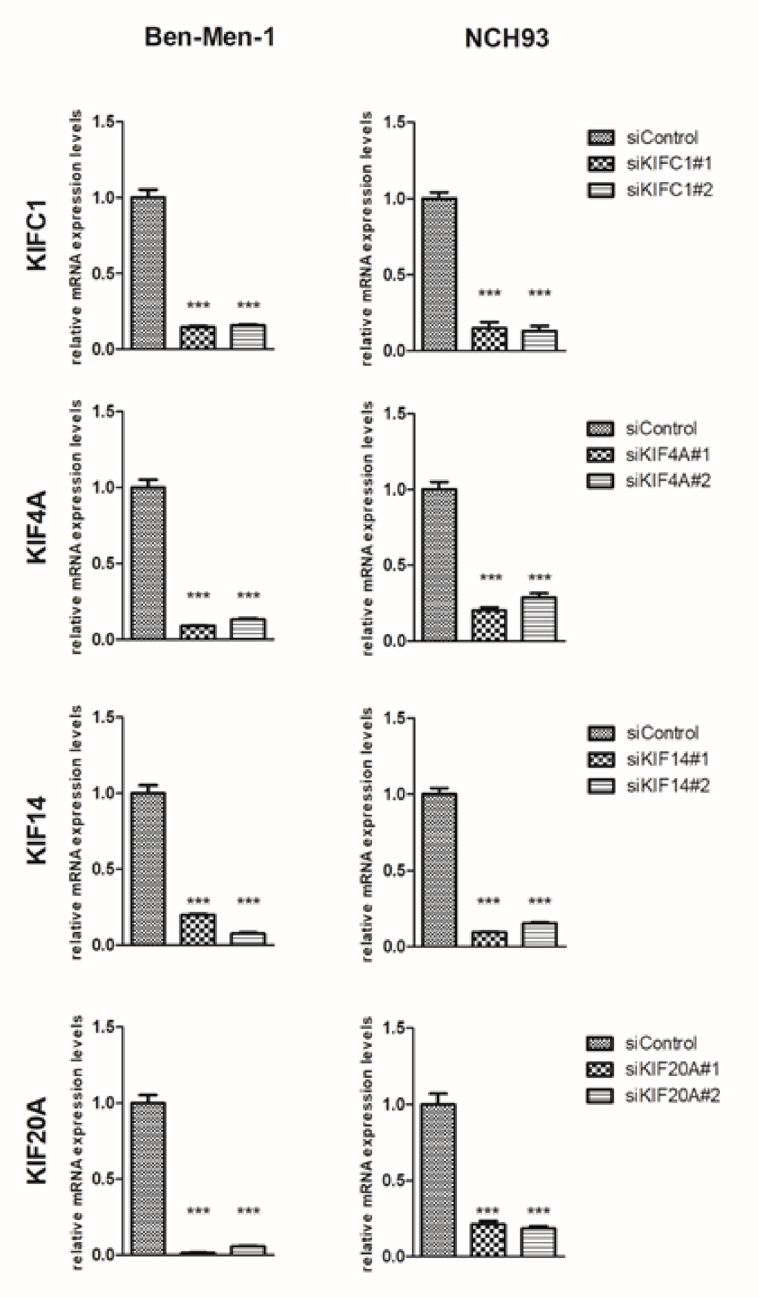

Figure S1. Efficiency of siRNA-mediated knockdown evaluated by qRT-PCR. Ben-Men-1 and NCH93 cells were transfected with 25 nM siRNAs. On the next day, cells were harvested and RNA isolation was performed. Knockdown efficiency was evaluated by qRT-PCR. Data were normalized to control siRNA. *** $p < 0.001$.

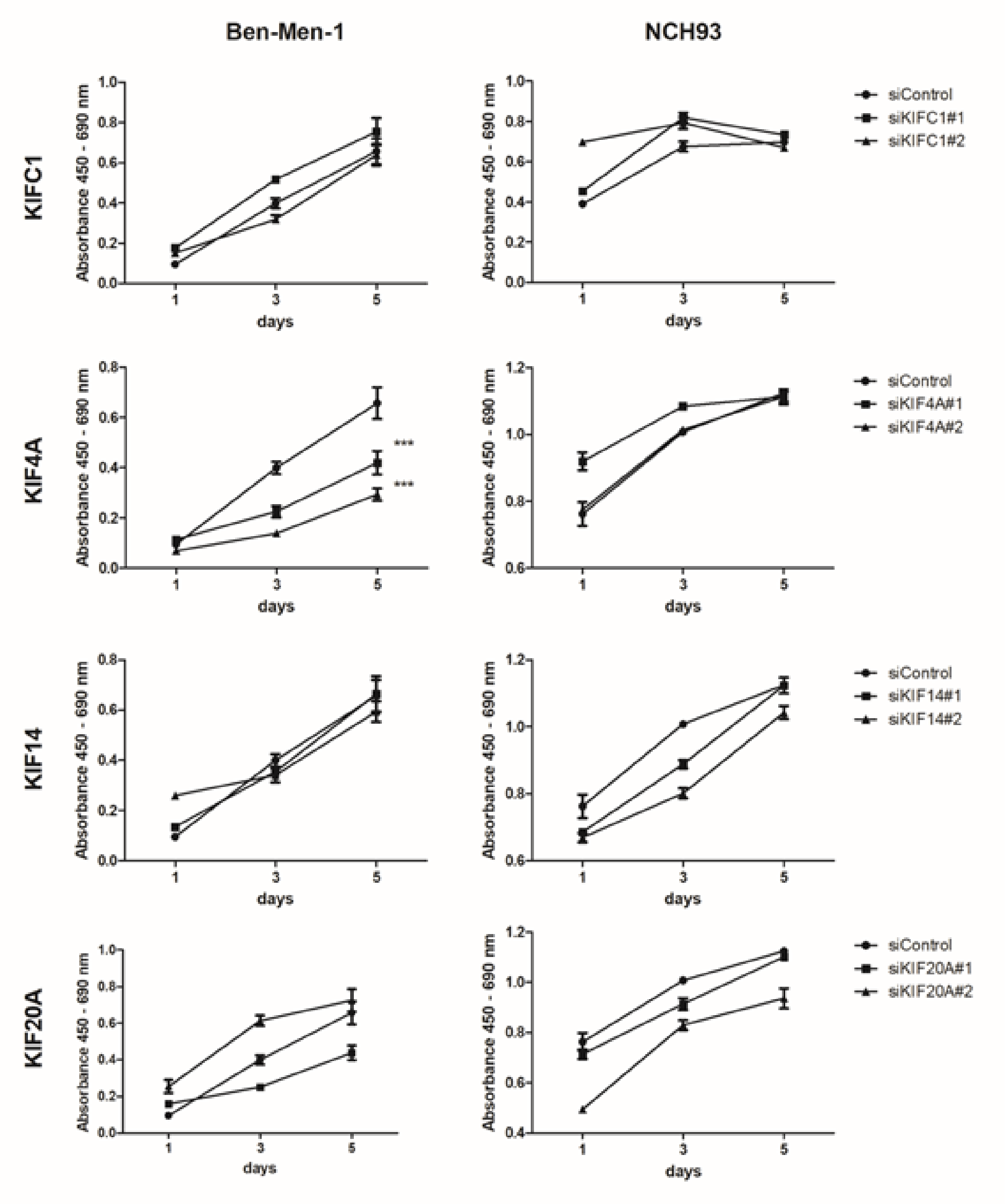

Figure S2. Knockdown of KIFC1, KIF4A, KIF14, and KIF20A failed to show a robust effect in both meningioma cell lines. Ben-Men-1 and NCH93 cells were transfected with 25 nM siRNAs. On the next day, 5000 cells were seeded in a 96-well plate. BrdU proliferation assay was performed on day 1, day 3 and day 5 after seeding.

Table S1. Primer sequences.

Gene Name	Sequence 5' to 3'
KIFC1	Forward GAAGAACGGAGGGGACTGAT<br>Reverse ACAGGGCTGCTTCTGATGTC
KIF4A	Forward GCGCCACTCATAAAAGGTGTA<br>Reverse GCACCTCCCATTGAATAGGTT
KIF11	Forward CATCCAGGTGGTGGTGAGAT<br>Reverse TATTGAATGGGCGCTAGCTT
KIF14	Forward CCTGTCTTTTTGCTTATGGTCAG<br>Reverse TCTTCACTAAATCCCATCATCG
KIF20A	Forward CGGCGACTAGGTGTGAGTAAG<br>Reverse GGATCCCTTGCGACATGA

Table S2. Clinical data of meningiomas employed for protein expression analysis (*n* = 30).

Clinical Factors	Group	Patients	
		n	(%)
**Sex**	Male	11	37
	Female	19	63
**Age** at 1st diagnosis (years)	Median	60	
	Range	24–85	
**WHO Grade**	WHO°I	10	33.3
	WHO°II	10	33.3
	WHO°III	10	33.3
**Subtype**	Fibroblastic	4	13
	Meningothelial	3	10
	Transitional	2	7
	Atypical	10	33
	Anaplastic	6	20
	Rhabdoid	0	0
	Papillary	2	7
	Mixed/Unknown	3	10
**Location**	Convexity	0	0
	Falx	11	37
	Tentorial or parasagittal	4	13
	Cranial base	4	13
	Multiple	7	23
	Other/NA	5	17
**Primary or recurrent tumor**	Primary tumor	24	80
	Recurrent tumor	4	13
	Unknown	2	7
**Resection grade**	Simpson °I	7	23
	Simpson °II	12	40
	Simpson °III	4	13
	Simpson °IV	5	17
	Simpson °V	0	0
	Unknown	2	7
**Postoperative treatment**	Radiotherapy	15	50
	Chemotherapy	1	3

Table S3. Comparative expression of kinesin family members in a published microarray dataset [38].

(A)

Gene	Fold-Change	*p*-Value	Fold-Change	*p*-Value	Fold-Change	*p*-Value
	**1R vs 1NR**		**2R vs 1NR**		**3 vs 1NR**	
KIFC1	1.56	2.64×10^{-4}	1.59	5.01×10^{-3}	1.96	2.20×10^{-7}
KIF4A	1.43	2.06×10^{-4}	1.49	6.69×10^{-4}	1.67	5.02×10^{-7}
KIF11	1.27	8.35×10^{-3}	1.52	3.53×10^{-3}	1.67	2.36×10^{-7}
KIF14	1.32	1.98×10^{-3}	1.41	1.73×10^{-4}	1.69	4.83×10^{-10}
KIF20A	1.64	2.12×10^{-3}	1.72	8.97×10^{-4}	2.22	4.93×10^{-9}

(B)

WHO°	Clinico-Pathological Subgroup	Definition	*n*
WHO °I	1NR	WHO °I without any further recurrence within the observation period (60 months)	13
	1R	WHO °I tumor recurring during the observation period (60 months)	7
WHO °II	2NR	WHO °II without any further recurrence within the observation period period (60 months)	6
	2R	WHO °II tumor recurring during the observation period (60 months)	8
WHO °III	3NR	WHO °III without any further recurrence within the observation period period (60 months)	4
	3R	WHO °III tumor recurring during the observation period (60 months)	16
	3NA	WHO °III with no available clinical data	8

Table S4. Panel Sequencing data of NCH93 cells.

Gene	Nucleotide Substitution	Exon	Amino Acid Change	Type of Mutation	COSMIC Data	FATHMM-MKL Score	FATHMM-MKL Prediction
**ALK**	T1787A	9	M596K	missense	NA		
	A4381G	29	I1461V	missense	NA		
	A4472G	29	K1491R	missense	COSM1130802	0.92	pathogenic
	C4587G	29	D1529E	missense	NA		
**APC**	T5411A	14	V1804D	missense	NA		
	T5465A	16/17	V1822D	missense	NA		
**ATM**	A5948G	40	N1983S	missense	NA		
**ATR**	T632C	4	M211T	missense	COSM149487	0.11	neutral
	G891C	4	K297N	missense	NA		
**BRCA1**	A1525G	14	S509G	missense	NA		
	G1936A	9	D646N	missense	NA		
	G2077A	10	D693N	missense	NA		
	C2471T	9	P824L	missense	NA		
	C2612T	10	P871L	missense	COSM148278	0.02	neutral
	A2972G	9	E991G	missense	NA		
	A3113G	10	E1038G	missense	NA		
	A3407G	9	K1136R	missense	NA		
	A3548G	10	K1183R	missense	COSM148277	0.02	neutral
	A4696G	14	S1566G	missense	NA		
	A4837G	15	S1613G	missense	NA		
	A4900G	16	S1634G	missense	NA		
**BRCA2**	A1114C	10	N372H	missense	COSM147663	NA	
	T7397C	14	V2466A	missense	NA		
	G8182A	18	V2728I	missense	NA		
**D2HGDH**	G164A	2	R55Q	missense	NA		
	G610A	7	V204I	missense	NA		
	G1012A	8	V338I	missense	NA		
**EGFR**	G1562A	13	R521K	missense	NA		
	T2189G	19	L730R	missense	NA		
**EPS8L3**	C877T	10	H293Y	missense	NA		
	C880T	10	H294Y	missense	NA		
**FGFR4**	C407T	3	P136L	missense	NA		
	G1162A	9	G388R	missense	COSM1567768	NA	
**FLT3**	C680T	6	T227M	missense	NA		
**GNAS**	T611C	7	F204S	missense	NA		
	T614C	7	F205S	missense	NA		
	T656C	8	F219S	missense	NA		
	T659C	8	F220S	missense	NA		
	T2585C	8	F862S	missense	NA		
**IDO2**	C742T	9	R248W	missense	NA		
	T1077A	11	Y359X	nonsense	NA		
**KLK1**	G433C	3	E145Q	missense	NA		
	A556G	4	K186E	missense	NA		

**KMT2D**	C2438T	10	P813L	missense	NA		
	G15566T	48	G5189V	missense	NA		
**MPL**	G340A	3	V114M	missense	NA		
**NBN**	G553C	5	E185Q	missense	NA		
**NF2**	390delC	5	D130fs	frameshift deletion			
	516delC	6	D172fs	frameshift deletion			
	639delC	7	D213fs	frameshift deletion			
**NOTCH2**	C57G	1	C19W	missense	COSM132738	0.33	neutral
**PCDH8**	T2228C	1	V743A	missense	NA		
	A1099G	1	T367A	missense	NA		
**PIK3CA**	A1173G	7	I391M	missense	COSM328028	NA	
**PIK3C2G**	384_386del	2	128_129del	nonframeshift deletion			
**PTCH1**	C3491T	23	P1164L	missense	NA		
	C3746T	23	P1249L	missense	NA		
	C3941T	23	P1314L	missense	NA		
	C3944T	23	P1315L	missense	COSM1638394	1.00	pathogenic
**SETD2**	C5885T	12	P1962L	missense	COSM149376, COSM149375	NA	
**TCF4**	C32T	1	A11V	missense	NA		
	G28C	1	A10P	missense	NA		
**TP53**	C98G	3	P33R	missense	NA		
	C215G	4	P72R	missense	COSM250061	0.36	neutral
